# Usefulness of hepatobiliary scintigraphy for predicting late complications in patients with choledochal cysts

**DOI:** 10.1007/s00383-022-05241-9

**Published:** 2022-09-20

**Authors:** Yunosuke Kawaguchi, Keita Terui, Shugo Komatsu, Mitsuyuki Nakata, Ryohei Shibata, Hiroko Yoshizawa, Tomoya Hirokawa, Erika Nakatani, Tomoro Hishiki

**Affiliations:** Department of Pediatric Surgery, Graduate School of Medicine, Chiba University, 1-8-1 Inohana, Chuo-ku, Chiba, 260-8677 Japan

**Keywords:** Cholestasis, Choledochal cyst, Hepatobiliary scintigraphy, Late complications

## Abstract

**Purpose:**

Hepatobiliary scintigraphy is a minimally invasive imaging method that evaluates bile flow dynamics. At our hospital, it has been performed for postoperative evaluation of patients with choledochal cysts (CC). This study evaluated the usefulness of biliary scintigraphy for predicting late complications in patients with CCs.

**Methods:**

The study included pediatric patients with CC who underwent surgery at Chiba University Hospital from 1978 to 2020, followed by postoperative biliary scintigraphy and subsequent radiologic evaluation. The patients were divided into two groups according to the presence or absence of “biliary cholestasis” on biliary scintigraphy.

**Results:**

The study included 108 patients, with a median age at surgery of 2 years and 11 months. The median follow-up period was 5203 days, with 11 hepatolithiasis cases and 8 cholangitis cases. No patients had cholangiocarcinoma. Twelve patients were considered to have “cholestasis” following biliary scintigraphy evaluation. There was no significant difference in the occurrence of hepatolithiasis between the cholestasis and non-cholestasis groups (*p* = 0.47), but cholangitis was significantly more common in the cholestasis group (*p* = 0.016).

**Conclusion:**

Biliary cholestasis on postoperative hepatobiliary scintigraphy was a risk factor for cholangitis in patients with CCs. These particular patients should be monitored carefully.

## Introduction

Choledochal cyst (CC) is a congenital biliary disease involving focal dilatation of the extrahepatic bile ducts [1]. CCs are typically associated with pancreaticobiliary maljunction, wherein the biliary and pancreatic ducts converge outside the sphincter of Oddi [2]. CCs cause various pathological conditions of the biliary tract and pancreas, such as biliary tract cancer, cholangitis, and pancreatitis. Biliary tract cancer predominantly occurs in the dilated bile duct and gallbladder [3]. Therefore, standard surgical procedures involve extrahepatic bile duct resection, including the gallbladder, which is the principal site of carcinogenesis, and biliary tract reconstruction [1]. This surgical strategy, which includes cyst excision and hepaticojejunostomy, has been established and results in good prognoses [4]. However, patients with CCs can develop late postoperative complications, such as cholangitis, hepatolithiasis, and residual cholangiocarcinoma [5, 6]. Postoperative cholangitis and hepatolithiasis are thought to be caused by cholestasis (bile stasis) and have been reported to occur more frequently in patients with a CC Todani classification of IV-A [7]. However, no objective method has been established to evaluate biliary flow after CC surgery.

^99m^Tc-*N*-pyridoxyl-5-methyltryptophan (^99m^Tc-PMT) hepatobiliary scintigraphy is a minimally invasive diagnostic imaging method that physiologically evaluates bile flow dynamics. It has been used for the postoperative evaluation of cholangiocarcinoma surgery in adults and liver transplantation in children [8–10]. However, the usefulness of ^99m^Tc-PMT hepatobiliary scintigraphy after pediatric CC surgery has not been systematically evaluated. Therefore, this study aimed to evaluate the usefulness of postoperative ^99m^Tc-PMT hepatobiliary scintigraphy for predicting late complications in patients with CCs.

## Patients and methods

### Patients

This retrospective observational study included 157 pediatric patients with CCs who underwent corrective surgery at Chiba University Hospital from 1978 to 2020, 139 of whom underwent ^99m^Tc-PMT hepatobiliary scintigraphy postoperatively. In total, 108 of the 157 patients whose images were evaluated by certificated radiologists were included. Patients’ data, including sex, age at the time of surgery, biliary dilatation classification (Todani classification) [7], ^99m^Tc-PMT hepatobiliary scintigraphy imaging findings, late complications (cholangitis, hepatolithiasis, and cholangiocarcinoma), and medical interventions for late complications, were collected. Late complications were defined as complications occurring 3 months or more postoperatively [11].

### Surgical procedure

Our reconstruction strategy was the following. The jejunum was divided 20 cm distal to the ligament of Treitz, and the proximal portion of the jejunum was anastomosed to the mid-jejunum 40 cm distal to the hepaticojejunostomy. Most patients underwent end-to-end anastomosis; however, if this was not possible, patients underwent side-to-end anastomosis. Intraoperative cholangiography and cholangioscopy were used to identify intrahepatic bile duct strictures. The anastomosis was performed without a stent and via the retrocolic route.

### ^99m^Tc-PMT hepatobiliary scintigraphy

**Fig. 1:**
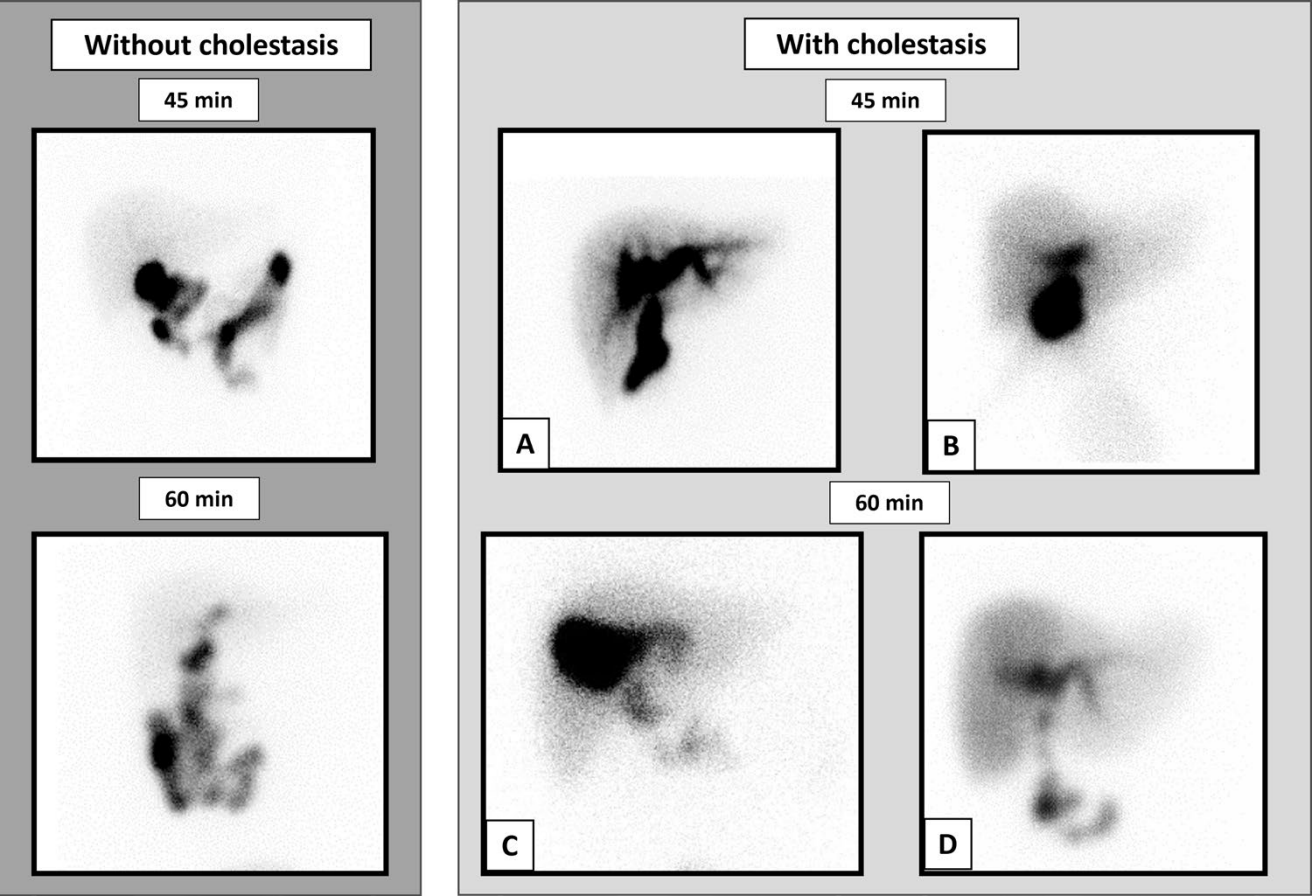
Typical images of cholestasis and non-cholestasis on ^99m^Tc-N-pyridoxyl-5-methyltryptophan hepatobiliary scintigraphy. With cholestasis: Upper row: Nuclide excretion is not observed beyond the Roux-en-Y anastomosis into the intestinal tract at 45 min after injection. **A**: Dilatation of the intrahepatic bile ducts and no nuclide excretion beyond the Roux-en-Y anastomosis. **B**: Dilatation of the intrahepatic bile ducts and Roux-en-Y anastomosis. Lower row: Nuclide remains within the liver; this is clearly delineated at 60 min after injection. **C**: Nuclide remains in the intrahepatic bile ducts and liver. **D**: Nuclide is retained within the right side of the liver

^99m^Tc-PMT hepatobiliary scintigraphy was performed when the patient was in good general condition and asymptomatic after the operation. Each patient received an intravenous injection of ^99m^Tc-PMT and was evaluated based on visual interpretation at 45 and 60 min after injection. We defined “cholestasis” as either the absence of nuclide excretion beyond the Roux-en-Y anastomosis into the intestinal tract at 45 min after injection or the clear delineation of retained nuclide within the liver at 60 min after injection (Fig. 1) [10, 12].

### Evaluation of the diagnostic capability of ^99m^Tc-PMT hepatobiliary scintigraphy

We divided the patients into biliary cholestasis and non-biliary cholestasis groups according to the ^99m^Tc-PMT hepatobiliary scintigraphy findings and compared the cumulative incidence of late complications (cholangitis, hepatolithiasis, and cholangiocarcinoma) after CC surgery.

### Statistical analysis

Continuous variables were expressed as the median (range, minimum to maximum). We also used the Mann–Whitney *U* test to compare the parameters and the two-tailed Fisher’s exact test to compare categorical data. Because the follow-up postoperative period varied from case to case, a log-rank test was used to compare the two groups. Statistical significance was set at *p* < 0.05. Statistical analyses were performed using the EZR software program [13].

## Results

### Clinical features of patients with CCs

The study participants included 26 men (20%) and 82 women (80%). Their CC Todani classifications were Ia (28/108, 26%), Ic (34/108, 31%), II (2/108, 2%), and IV-A (44/108, 41%). All patients underwent extrahepatic bile duct resection encompassing the gallbladder and hepaticojejunostomy (Roux-en-Y). Of the included patients, 105 underwent open surgery, and 3 underwent laparoscopy. The median age at the time of surgery was 2 years and 11 months (range 1 month–15 years and 10 months). The median follow-up postoperative period was 5203 days (range 183–15,720 days). There were 34 patients (32%) with a follow-up period of less than 10 years, 38 patients (35%) with a follow-up period of 10–20 years, and 36 patients (33%) with a follow-up period of more than 20 years. The median postoperative day of ^99m^Tc-PMT hepatobiliary scintigraphy imaging was 23 days (range 8–3,517 days).

There were 11 cases (11%) of hepatolithiasis and 8 cases (7.4%) of cholangitis (including overlaps). No patients were diagnosed with cholangiocarcinoma at the time of primary surgery or in the follow-up period. The median postoperative periods at the time of complication diagnosis were 6,249 days (range 1,401–12,381 days) for hepatolithiasis and 6,844 days (range 180–10,442 days) for cholangitis. Including overlaps, seven cases (7/11, 64%) of hepatolithiasis and 3 cases (3/8, 38%) of cholangitis required intervention later (Tables 1 and 2).

**Table 1:** Characteristics and late-stage complications of patients with choledochal cysts

Patients, total *n* = 108	*n* (%)
Sex, *n* (%)	
Male	26 (20)
Female	82 (80)
Todani classification, *n* (%)	
Ia	28 (26)
Ic	34 (31)
II	2 (2)
IV-A	44 (41)
Surgical procedure	
Open	105 (97)
Laparoscopic	3 (3)
Age at surgery*	2 years 11 months (range 3 months–15 years 10 month)
Follow-up postoperative days*	5203 (range 183–15,720)
Follow-up postoperative period	
< 10 years	34 (32)
10–20 years	38 (35)
> 20 years	36 (33)
Postoperative days of hepatobiliary scintigraphy*	23 (range 8–3517)
Late-stage complication, *n* (%)	
Hepatolithiasis	11 (10)
Postoperative days at diagnosis of hepatolithiasis*	6249 (range 1401–12,381)
Cholangitis	8 (7)
Postoperative days at diagnosis of cholangitis *	6844 (range 180–10,442)
Cholangiocarcinoma	0 (0)

*Median

**Table 2:** Late-stage complications and interventions

No	Follow-up^*1^	Age at surgery^*2^	Todani classification	Hepatolithiasis	POD^*3^	Cholangitis	POD^*4^	Intervention
1	15,910	02/05	Ic	+	15,544	–		DBE^*5^
2	14,549	09/07	IV-A	+	7995	+	7995	DBE^*5^
3	14,544	03/01	Ia	–		+	10,442	–
4	13,291	01/10	IV-A	+	12,381	–		PTCS^*6^
5	12,837	00/03	Ia	–		+	8787	–
6	11,214	03/02	Ia	–		+	7615	–
7	10,605	04/05	IV-A	+	10,309	–		–
8	10,556	00/05	Ia	+	4893	–		Left lobectomy re-anastomosis
9	10,025	01/12	IV-A	+	6427	–		–
10	9114	04/03	IV-A	+	1497	–		–
11	7292	15/10	Ia	+	2005	+	2128	PTCS^*6^
12	6585	01/02	IV-A	-		+	1815	–
13	6319	01/06	IV-A	+	6249	+	6072	–
14	6173	02/08	IV-A	+	3604	–		Left lobectomy
15	1555	09/05	I-A	+	1401	–		–
16	642	13/10	IV-A	–		+	180	DBE^*5^

^*^1 Follow-up: follow-up postoperative days

^*^2 Age at surgery: year/month

^*^3 POD: postoperative days at the time of diagnosis of hepatolithiasis

^*^4 POD: postoperative days at the time of diagnosis of cholangitis

^*^5 DBE: double-balloon enteroscopy and balloon dilatation

^*^6 PTCS: percutaneous transhepatic cholangioscopy

### Usefulness of ^99m^Tc-PMT hepatobiliary scintigraphy for patients after CC surgery

Focusing on biliary cholestasis on ^99m^Tc-PMT hepatobiliary scintigraphy, we compared cases in the cholestasis and non-cholestasis groups. There were 12 cases of cholestasis. There were no significant differences between the cholestasis and non-cholestasis groups in terms of sex (2 vs. 24 male patients, respectively, *p* = 0.73), number of patients with Todani classification IV-A (7 vs. 37 cases, respectively, *p* = 0.22), laparoscopic procedures (1 vs. 2 cases, respectively, *p* = 0.3), age at the time of surgery (1,061 vs. 1,055 days, respectively, *p* = 0.91), and the postoperative day on which ^99m^Tc-PMT hepatobiliary scintigraphy imaging was performed (20 vs. 25 days, respectively, *p* = 0.55) (Table 3).

**Table 3:** Characteristics of the cholestasis and non-cholestasis groups

	With cholestasis	Without cholestasis	*p* value
	*n* = 12	*n* = 96	
Sex			
Male	2	24	0.73
Female	10	72	
Todani classification			
IV-A	7	37	0.22
Other	5	59	
Ia	3	5	
Ic	2	31	
II	0	3	
Surgical procedure			
Open	11	94	0.30
Laparoscopic	1	2	
Age at surgery (days)	1061 (505–1662)	1055 (532–1612)	0.91
Postoperative days of scintigraphy*	20 (13–73)	25 (13–86)	0.55

Median (interquartile range)

*Scintigraphy: ^99m^Tc-N-pyridoxyl-5-methyltryptophan hepatobiliary scintigraphy

**Fig. 2:**
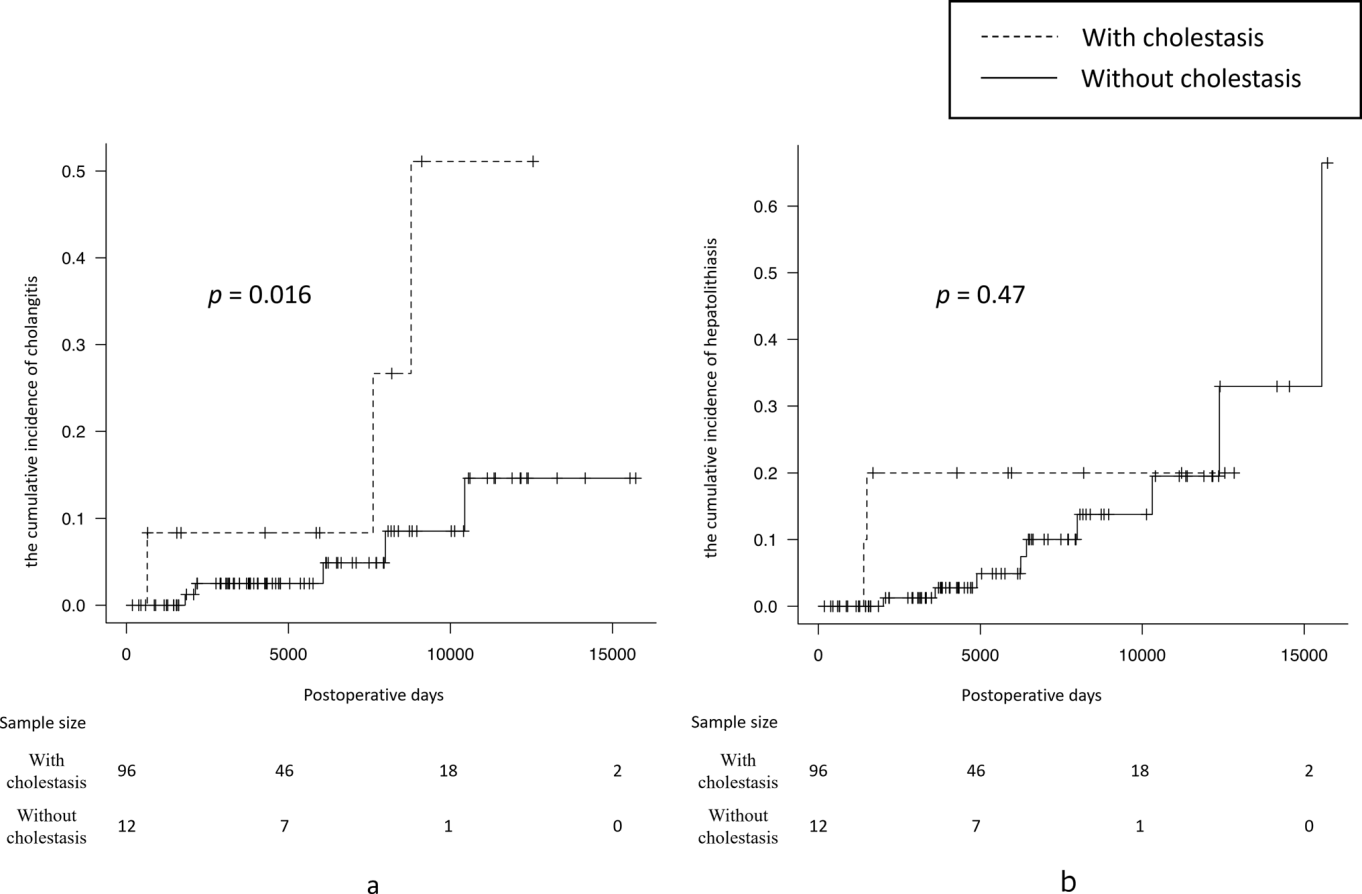
Cumulative incidence of cholangitis (**a**) and hepatolithiasis (**b**) in the cholestasis and non-cholestasis groups

Cholangitis was more prevalent in the cases with cholestasis (3/12, 25%) than in those without cholestasis (5/96, 5%). A log-rank test showed that cholangitis was significantly more common in the cholestasis group (*p* = 0.016) (Fig. 2a). There was no significant difference in the incidence of hepatolithiasis between the two groups (cholestasis: 2/12, 17% vs. non-cholestasis: 9/96, 9%; *p* = 0.47) (Fig. 2b). The finding of cholestasis was prognostic for cholangitis postoperatively, with a sensitivity of 0.38 and a specificity of 0.91. Three of the 8 cases of cholangitis required intervention later, one of which showed cholestasis on scintigraphy. The remaining 2 patients did not have cholestasis.

## Discussion

The principal finding of our study, in which we assessed the usefulness of postoperative 99mTc-PMT hepatobiliary scintigraphy for predicting late complications among patients with CCs, was that cholestasis at postoperative hepatobiliary scintigraphy appeared to be predictive of the subsequent occurrence of the late complication of cholangitis.

The incidence rate of cholangitis after biliary dilatation is reportedly 5–13% [6, 14, 15]. In the present study, the incidence rate of cholangitis was 7%, which was not significantly different from that in previous reports. Furthermore, the incidence rate of postoperative hepatolithiasis, albeit with variation between sites, is reportedly 2.7–10.7% [16–20]. However, according to our findings, the incidence rate of hepatolithiasis was 11%, which was slightly higher than that in previous reports. We believe that this was because many of our patients (69%) had a postoperative follow-up period exceeding 10 years. According to a report by Mukai et al., hepatolithiasis is more prevalent among patients who had surgery more than 10 years previously, indicating that the follow-up period has an impact on the incidence rate [20]. Many cases of hepatolithiasis require interventions, such as hepatectomy or percutaneous transhepatic cholangioscopy, and early discovery and treatment are considered beneficial.

Biliary cholestasis is a primary concern following surgery for a CC because cholestasis may cause hepatolithiasis [21] and/or cholangitis [22]. Hence, some reports recommend removal or restructuring of the stenosis during the initial surgery [23, 24], while others mention that making incisions in the left and right hepatic ducts reduces the risk of cholangitis [15]. However, Chijiiwa et al. performed percutaneous transhepatic cholangioscopy in patients with hepatolithiasis and reported no obvious bile duct stenosis [17]. Therefore, the cause of biliary cholestasis is not only anastomotic stenosis and intrahepatic bile duct stenosis but also dysfunction of the Roux-en-Y. Indeed, “biliary cholestasis” was diagnosed at scintigraphy in the case of either an absence of nuclide excretion beyond the Roux-en-Y anastomosis (an indicator of anastomotic dysfunction) or a clear delineation of the retained nuclide in the liver (an indicator of anastomotic stenosis or intrahepatic bile duct stenosis). The association between the type of biliary cholestasis and late complications remains a subject for future study.

In biliary tract surgery, the passage through the bile duct anastomosis may be evaluated postoperatively. In adults, hepatobiliary scintigraphy is used to evaluate bile excretion after surgery for biliary tract cancer [9, 10, 25]. Furthermore, the reliability of biliary stenosis diagnosis following liver transplantation in children is reportedly improved by the use of combined ultrasonography and biliary scintigraphy [8]. One description of a CC case reported that biliary scintigraphy could be used to diagnose cholestasis and elucidate the cause of symptoms in patients who developed postoperative cholangitis. In that case, ultrasonography showed no intrahepatic bile duct dilatation and no particular suggestion of bile duct stenosis, and the evaluation of cholestasis by biliary scintigraphy was pivotal for diagnosis [9]. In addition, a report by Mukai et al. also described a case in which biliary scintigraphy was performed for the evaluation of hepatolithiasis after CC, and although there were signs of cholestasis, the patient was not diagnosed with stenosis [20]. Although evaluation by biliary scintigraphy after CC surgery has reportedly been useful, such literature is only at the case report level, and our report is the first to evaluate a large sample of CC cases with biliary scintigraphy. Our report suggests that the occurrence rate of cholangitis is high among patients diagnosed with cholestasis using biliary scintigraphy. This suggests that evaluating cholestasis with biliary scintigraphy after CC surgery will allow surgeons to predict the likelihood of developing cholangitis in the future.

In this study, there were no cases of biliary tract cancer among the follow-up patients, and it was not possible to directly evaluate the effectiveness of biliary tract scintigraphy for biliary tract cancer post-CC surgery. However, since a history of postoperative cholangitis has been reported as a risk factor for developing biliary tract cancer [12, 26], it may be possible to evaluate the risk of biliary tract cancer after CC surgery using biliary scintigraphy.

Late complications may occur after CC surgery; hence, the importance of long-term follow-up has been suggested [4, 5]. A study by de Kleine et al. advocated conducting outpatient carbohydrate antigen 19–9 blood tests and liver ultrasonography every 6 months for 2 years after surgery, then performing lifelong follow-ups every 2 years [6]. Mukai et al. have mentioned the importance of educating patients about the possibility of late complications [20]. In their report, patients who were over the age of 18 years and continuing outpatient follow-up accounted for less than 20% of all postoperative patients, and self-interruption of hospital visits is the likely cause of the low visit rate. This is because pediatric surgeons rarely describe late complications to patients, many of whom are thus not aware of the need to continue hospital visits. By evaluating postoperative bile outflow using biliary scintigraphy, patients with a high risk of late postoperative complications can be determined, and more efficient patient follow-up can be performed. It would also be an effective tool for patient education. In contrast, many papers do not specify the follow-up methods and duration. To establish an appropriate postoperative follow-up strategy, it is necessary to continue to accurately ascertain the long-term follow-up data.

There are limitations to this study. First, as this is a retrospective study, there is the risk of bias with regard to the selection of tests and the end of follow-up. Second, as this is a long-term study, we cannot rule out the impact of procedure-linked bias on the outcomes. Third, biliary scintigraphy was performed at different postoperative times. Fourth, our analysis was qualitative, and we have not been able to carry out a quantitative assessment. Therefore, we believe that late complications can be predicted with greater accuracy by examining the localization and dynamics of abnormal nuclide accumulation in biliary scintigraphy in detail, and this requires further prospective study. Finally, although we showed the trend that biliary cholestasis ^99m^Tc-PMT hepatobiliary scintigraphy was a risk factor for cholangitis, further study is needed regarding its clinical application.

## Conclusion

Biliary cholestasis on postoperative ^99m^Tc-PMT hepatobiliary scintigraphy is a risk factor for cholangitis during follow-up of patients with CCs. These patients should be followed up carefully.
